# Progress in Nerve Surgery

**Published:** 1903-10-17

**Authors:** 


### PROGRESS IN NERVE SURGERY.

(*Concluded from page* 35.)

**The Necessity of Shortening Large Nerves when Amputating.**[2]—Spencer draws attention to the importance of always drawing out the large nerve trunks on the surface of a stump and cutting off a piece. He quotes a number of cases where this procedure had been omitted and where painful stumps resulted, the nerves in all the cases being adherent in the scar tissue. They were all cured by either dissecting out the nerve or amputating higher up. In the latter case the nerve was drawn out as suggested.

**The Blood-pressure Re-action of Acute Cerebral Compression Illustrated by cases of Intracranial Hæmorrhage.**[3]—Cushing contributes this paper to demonstrate in clinical cases the reactions corresponding to some experimental observations presented in the Mütter lecture.[4] He adopts Kocher's classification of the stages between the first onset of compression symptoms and the terminal paralysis. Stage I.: That of circulatory compensation corresponding to a mild degree of compression, during which the encroachment on the intracranial space is compensated for by the escape of cerebro-spinal fluid and narrowing of the venous channels. The symptoms are insignificant. Stage II.: Failure of this circulatory compensation and presence of a condition of venous stasis with lessening of amount of blood passing through capillaries. Symptoms of disturbed cerebral function—headache, vertigo, restlessness, excitement, or delirium, etc. Some distension and tortuosity of the retinal veins. The venous congestion is influencing the bulbar circulation, as evidenced by slowing of the pulse with or without a rise in blood pressure. Stage III.: That of advanced compression, corresponds to a condition of capillary anæmia due to further increase in intracranial tension. There may be a rise in blood pressure with respiratory disturbances and slow pulse. Stage IV.: The terminal one follows on an increase of intracranial tension over and above that which can be compensated for by stimulation of the vasomotor centre. An irrecoverable condition of cerebral anæmia has been reached. There is falling blood pressure, complete muscular relaxation, wide pupils, deep coma, etc. Five clinical cases are described at length, illustrating these four different stages, four of them being fractures of the skull with intracranial hæmorrhages and varying degrees of increase in the intracranial tension, and one a case of apoplexy. The blood pressure was taken in all these cases by a special apparatus, and it was found to correspond to the clinical signs indicated in the above four stages, rising in the first three and falling in the fourth stage. A brief summary of the paper brings out the following points:—Varying degrees of rapid increase in the intracranial tension produce corresponding disturbances in the intracranial circulation. To these the symptoms of compression are solely due, and are divisible into the four stages indicated above. The major symptoms originate in the centres in the medulla, and are only called out when the intracranial tension begins to approach the arterial tension, so that anæmia is threatened, this anæmic condition stimulating the vasomotor centre and causing a rise in blood pressure. Up to a certain point the extent of this rise may be taken as an indication of the degree of compression, beyond this the reaction fails. The practical outcome of the paper is that when taken in conjunction with other symptoms there is a progressive increase in arterial pressure, or a pressure which exhibits great alteration from moment to moment, early operation should be undertaken, and if there be no localising symptoms a large osteoplastic flap should be raised from one or other hemisphere to relieve the intracranial pressure.

**Multiple Sarcomatosis of the Central Nervous System.**—Spiller and Hendrickson[5] after pointing out that this disease has not received the attention it should, relate at great length three cases, a brief summary of which is as follows :—Case 1. A woman well until a year after marriage. Three days after birth of child severe occipital headache. Cessation of headache followed by dizziness and diplopia. Lower limbs gradually became stiff and weak, and finally paralysed. Control over bladder and rectum lost. Paralysis of both external recti and one oblique muscle, swelling of optic discs and tortuosity of vessels. Five months after birth of child, head slightly retracted and neck stiff, headache severe, lower limbs paralysed for sensation and motion, ankle clonus on right and Babinski's reflex on both sides. A large sarcoma was found at the autopsy in the left cerebellar lobe, two intramedullary sarcomata in the thoracic region of the spinal cord and numerous small sarcomata in the spinal pia. Case 2: A man aged 42. About a year before admission he began to have failure of eyesight and constant pain in left occipital region. Six months later he had difficulty in walking and would get giddy and fall. Had two attacks of unconsciousness, but without convulsions; also had attacks of vomiting. Labyrinthine deafness on both sides. Choked disc in the right eye and optic atrophy in the left. Both external recti paralysed, occasional attacks of vertigo. An unsuccessful attempt was made to remove a cerebellar tumour. Post-mortem a tumour was found in each cerebello-pontine angle, others about the Gasserian ganglia, pituitary body, floor of fourth ventricle, right internal auditory meatus and right jugular foramen, and numerous small tumours on the pia of the spinal cord. Case 3: A man who three weeks before admission had pain in the lower thoracic region and girdle pain. The left lower limb became weak, followed by weakness in the right lower limb. Incontinence of urine and fæces. No pain in lower limbs, but they became completely paralysed and sensation was lost in those parts below a line passing two inches below the nipple. Tendon reflexes in lower limbs absent, but Babinski's reflex obtained on either side. At the autopsy a primary sarcoma was found within the spinal cord, just above the mid-thoracic region. The difficulty in diagnosing multiple sarcomatosis is great, as there may be extensive disease with but few symptoms. The masses grow in the pia about the spinal cord and nerve roots, specially those of the cauda equina and about the cranial nerves. They have little tendency to invade the spinal cord as in tuberculosis and syphilis and cause few clinical signs of their presence. The diagnosis of these cases is gone into at length in the paper, and it would appear that sarcomatous meningitis, as the authors suggest the disease might be called, is most liable to be confounded with syphilitic meningitis.

**Fracture Dislocation of the Cervical Spine without Paralysis.**[6]—An interesting case is reported from St. Thomas's Hospital of a man at 56, who fell down stairs a month before admission. He struck the back of his head on the wall at the bottom of the stairs and forced his head forwards, the chin being driven into the sternum. He got up and resumed his work next day, and continued to work for a fortnight afterwards. He complained only of a stiff neck and some pains at the back of the head and shoulders. The neck was held forwards stiffly and appeared to be thickened. There was *no paralysis*, but a skiagram showed a fracture through the body of the axis with the displacement of the body of that bone and the atlas forwards.

**Spasmodic Torticollis Relieved by Operation.**[7]—Abbe showed a patient who had had symptoms of spasmodic torticollis for about 13 years. She first came under his notice three years ago. Her head was then constantly drawn over the left shoulder, and could only be momentarily straightened with considerable effort. At first he resected the second posterior cervical nerve. This was followed by much improvement. A few months later, under cocaine, the tendon of the sterno-mastoid at its sternal attachment was divided. This was followed by marked and immediate improvement, which has continued up to the present time. The patient could now keep her head in the straight position without effort, and the trembling she previously suffered from had ceased. Dana and Bryant thought that the best results were obtained by dividing the muscles as well as resecting the nerves, and thought that resection of the spinal accessory alone was rarely followed by good results. Starr thought that as the disease was a central one it would be more rational to excise the cortical centre for movements of the head. None of the cases that he had operated on by division of nerves or muscles had been absolutely satisfactory.

[2]Brit. Med. Jour., July 11, 1903. [3] Amer. Jour. of Med. Science, June, 1903. [4] Ibid., Sept., 1902. [5] Ibid., July, 1903. [6] Lancet, July 25, 1903. [7] Med. Rec., July 4, 1903.

